# Distinct Characteristics of Small Cell Lung Cancer Correlate With Central or Peripheral Origin: Subtyping Based on Location and Expression of Transcription Factor TTF-1

**DOI:** 10.1097/MD.0000000000002324

**Published:** 2015-12-28

**Authors:** Eisaku Miyauchi, Noriko Motoi, Hiroshi Ono, Hironori Ninomiya, Fumiyoshi Ohyanagi, Makoto Nishio, Sakae Okumura, Masakazu Ichinose, Yuichi Ishikawa

**Affiliations:** From the Division of Pathology, The Cancer Institute, Department of Pathology, The Cancer Institute Hospital, Japanese Foundation for Cancer Research (JFCR) (EM, NM, HO, HN, YI); Thoracic Center, The Cancer Institute Hospital, JFCR, Tokyo (FO, MN, SO); and Department of Respiratory Medicine, Tohoku University Graduate School of Medicine, Sendai, Japan (EM, MI).

## Abstract

Small-cell lung carcinoma (SCLC) is a type of lung cancer with neuroendocrine differentiation and a poor prognosis that is widely believed to arise in the central lung. Thyroid transcription factor-1 (TTF-1) is a peripheral marker of lung adenocarcinoma that is also highly expressed in SCLC. In this study, we examined whether SCLC is really a central-type tumor and the relationship between tumor location, TTF-1 expression and prognosis of SCLC.

Ninety six SCLCs, diagnosed from biopsies or surgical materials, for which detailed computed tomography (CT) images were available, were collected consecutively from Japanese patients between 2004 and 2011. We examined the location of the primary tumor (central or peripheral) using thin-sliced CT, a TTF-1 immunohistochemical expression, and clinicopathology including prognosis.

Of the 96 SCLCs, 74% (71/96) were of the peripheral type and found to have a significantly worse prognosis than central-type tumors. TTF-1 immunoreactivity was identified in 79 tumors (82%), 78% of which (62/79) were of the peripheral type and 22% of which were central. TTF-1 expression was significantly correlated with peripheral location (*P* = 0.030). Multivariate analysis revealed that high TNM stages and the peripheral location were independent markers for poor survival.

The majority of SCLCs were of the peripheral type. The peripheral-type SCLC expressed TTF-1 more frequently and had a poorer prognosis than central-type tumors did. Further analysis on original sites of SCLC, using molecular methodology, or based on another ethnicity, should be warranted.

## Abstract 2

Supplemental Digital Content is available in the text

## INTRODUCTION

Lung cancer is a leading cause of cancer deaths worldwide. Despite recent improvements in treatment, the prognosis remains poor. Small cell lung carcinoma (SCLC), a type of lung cancer with neuroendocrine differentiation, accounts for 10 to 15% of all lung carcinomas.^1^ SCLC is more responsive to chemotherapy and radiation therapy than other types of lung cancer. However, its prognosis is the poorest among all histological types of lung cancer, with an overall survival rate at 5 years of only 5 to 15%.^2^

It has been thought that SCLC is a central-type lung cancer because it is usually observed at the central area of chest x-ray pictures when the tumor is first diagnosed. The long-standing notion that SCLC originates from the central lung may be based on such observations, rather than on close examination of whether SCLC really originates from the central lung. In fact, there are no previous reports about the detailed analysis of primary tumor location of SCLC by thin-sliced CT.

Thyroid transcription factor-1 (*TTF-1),* also known as *TITF1* or *Nkx2–1*, encodes a 38-kDa homeodomain-containing nuclear protein and was initially identified as an activator of thyroid-specific gene transcription.^3^ It is expressed in the thyroid, lungs, and brain.^4^ In the normal adult lung, the expression is restricted to type II alveolar cells and club (Clara) cells, which are found at the terminal respiratory unit (TRU).^5–7^ The expression is maintained in 62% to 76% of peripheral type adenocarcinomas.^5,8–10^ Therefore, TTF-1 is considered a marker of the TRU-type adenocarcinoma of the lung. In almost all other organs except thyroid, adenocarcinomas are negative for TTF-1. Because of its specific expression in lung adenocarcinomas, TTF-1 has been used as a diagnostic marker for primary and metastatic lung adenocarcinoma.^11^

TTF-1 is differentially expressed according to tumor types. In particular, reports indicate that as many as 81% to 97% of SCLCs express TTF-1, whereas it is never or rarely expressed in squamous cell carcinoma of the lung.^8–10,12–14^ Regarding small cell carcinoma in general, TTF-1 is expressed not only in SCLC but also in small cell carcinoma of other organs, although the frequency of expression varies.^12,15^ Therefore, concerning SCLC, TTF-1 is not necessarily a marker of the lung origin. Recently, enigmatic roles of TTF-1 have been noted: it appears to act as a “lineage-survival” oncogene and a protector against tumor progression in lung adenocarcinomas.^16,17^ These facts raise questions about the roles of TTF-1 in SCLC: Specifically, is TTF-1 a useful marker for SCLC location, or is TTF-1 expression a prognostic factor for SCLC?

In this study, to address these questions, we examined whether SCLC was really a central-type tumor and whether tumor location and TTF-1 expression have prognostic relevance, using thin-sliced chest CT images, TTF-1 expression data, and clinicopathological data including prognosis.

## MATERIALS AND METHODS

### Patients

**FIGURE 1:**
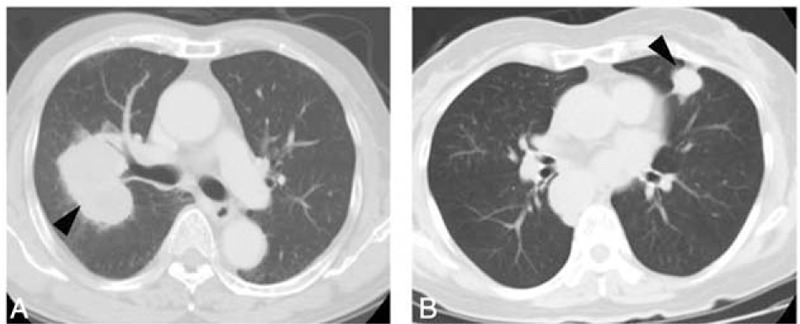
Typical thin-sliced computed tomography (CT) pictures of central (a) and peripheral (b) types of small cell lung carcinoma (SCLC). Each arrow indicates the primary mass. For definition of central and peripheral tumors, see the text. CT = computed tomography, SCLC =  small cell lung carcinoma.

A series of 96 consecutive patients with SCLCs diagnosed from biopsies (n = 78) or surgical materials (n = 18) between 2004 and 2011 at the Cancer Institute Hospital, Japanese Foundation for Cancer Research (JFCR), Tokyo, Japan, were enrolled in the present study. According to the Japan Lung Cancer Society Therapy Guideline,^18^ clinical stage I SCLC is surgically removed when patients’ performance status is good, and followed by adjuvant chemotherapy. All the other stage tumors are treated by chemotherapy and/or irradiation. Here, the tumors, for which only biopsy materials were available, were inoperable. SCLCs diagnosed only by cytology were excluded because of the difficulty of immunohistochemical evaluation. The hospital records for all cases were available and were reviewed to obtain clinicopathological variables such as age, sex, tumor size, TNM stage, and smoking history. All patients underwent multidetector CT imaging (2- to 5-mm section thickness) at the time of diagnosis and the location of primary tumor (central or peripheral) was evaluated by experienced diagnostic experts (FO, MN) and, in the case of split opinions, other physicians (EM, HO, and SO) who are routinely engaged in CT diagnosis were also involved in discussion with careful distinction of primary and metastatic tumors, leading to consensus. Based on previous reports,^5,19^ tumors involving segmental or more proximal bronchi were defined as a central type (Figure 1A), whereas tumors involving subsegmental or more distal bronchi were defined as a peripheral type (Figure 1B). A few cases with extensive atelectasis were excluded from this study because of the difficulty of determining the central/peripheral status. All patients included in this study provided informed consent for research and the study plan was approved by the institutional review board of JFCR (named JFCR IRB).

### Histology and Immunohistochemistry

**FIGURE 2:**
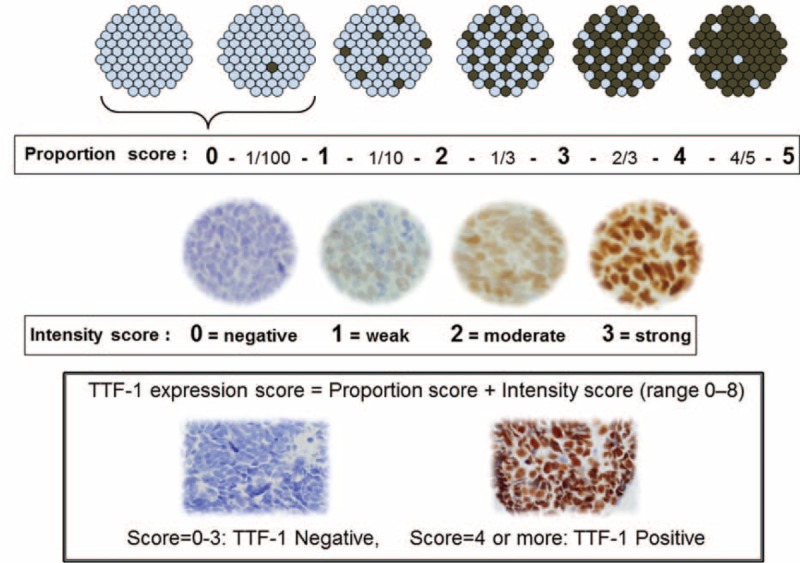
TTF-1 expression score was defined as a sum of the proportion score (0 to 5) and the intensity score (0 to 3). Score 0 to 3 was defined negative and Score 4 or more positive.

All hematoxylin-eosin stained slides derived from formalin-fixed, paraffin-embedded tissue specimens were reviewed, and the pathological diagnoses of SCLC were confirmed by pathologists (EM, NM, YI) including leading lung pathologists, according to the WHO criteria.^20^ In addition, neuroendocrine markers (synaptophysin, chromogranin-A, and NCAM), basal cell markers (CK5/6, K903 [CK34βE12], p40, p63) and Ki-67, as necessary, were used to determine the neuroendocrine and basal-cell natures of tumors and to assist with SCLC diagnosis. The expression of TTF-1 and other tumor markers was examined immunohistochemically using 4-μm-thick sections of paraffin-embedded tissue obtained via surgery or transbronchial/needle biopsy. For TTF-1 staining, the antibody clone 8G7G3/1 (Dako, Glostrup, Denmark) was used in optimized protocols on a Dako Autostainer (Dako, Glostrup, Denmark), and particular attention was paid to using appropriately fixed tissues and freshly prepared sections. There is another clone, SPT24 (Leica), and 8G7G3/1 is more specific and less sensitive than SPT24. Immunohistochemistry details are shown in Suppl. Table 1. To evaluate the immunohistochemical staining of TTF-1, only distinct nuclear staining of the tumor cells was considered. Type II pneumocytes and club (Clara) cells served as internal controls for the evaluation of antigen preservation. For evaluation of TTF-1 expression, sum of the Immunohistochemical staining was used on the basis of a proportion score (0–5) and an intensity score (0–3). Sum of them provides a TTF-1 expression score (0–8). These scores were then reviewed and a consensus was reached that Score 0–3 was defined negative and Score 4 or more positive. Details are shown in Figure 2.

### Statistical Analysis

The data were analyzed using the statistical software package IBM SPSS Statistics 19 (IBM, Tokyo, Japan). Overall survival was defined as the time between the date of diagnosis and the date of the last follow-up or death. Pearson's chi-square test and Fisher's exact test as appropriate were applied to examine the association between two categorical variables, TTF-1 expression and tumor location. The Kaplan–Meier method was used to estimate overall survival in each of the groups. The significance of differences in survival between the groups was determined using the log-rank test and Pearson's chi-square test as appropriate. To evaluate the independent prognostic relevance of tumor location and TTF-1 expression, the multivariate analysis using the Cox regression model was performed. The assumption of proportional hazard was confirmed using the time-dependent Cox regression model in the statistics software. Significance was defined as *P* < 0.05.

## RESULTS

The patients’ clinicopathological characteristics are summarized in Table 1. Eighteen (19%) of the 96 patients underwent surgery with adjuvant chemotherapy, and the others were treated only with chemo- and/or radiation therapy. The median age at diagnosis was 68 years old, and 84% of the patients were male. As expected, the large majority (98%) of the patients were ever smokers. Regarding clinical stages, 79% (76/96) were in stage III or IV. Pathological stages of cases with surgery were 1A (n = 6), 1B (n = 0), 2A (n = 5), 2B (n = 2), 3A (n = 4), 3B (n = 1) and 4 (n = 0). In the cases with biopsies, a CT-guided needle biopsy specimen was available for one case and, for all the others, transbronchical biopsy materials were used. In the cases undergoing surgery, partial resection was applied to one case and the others underwent lobectomy. The median follow-up period was 16.7 months (2.0–77.7 months). On CT images, 2 patients had 2 tumor nodules in the lung fields, and we postulated that the larger lesion was primary. No patients presented with three or more nodules, possibly because we excluded cases that were diagnosed only by cytology. We found that 12 patients had no lymph node metastasis and all those cases were of the peripheral type.

**TABLE 1:**
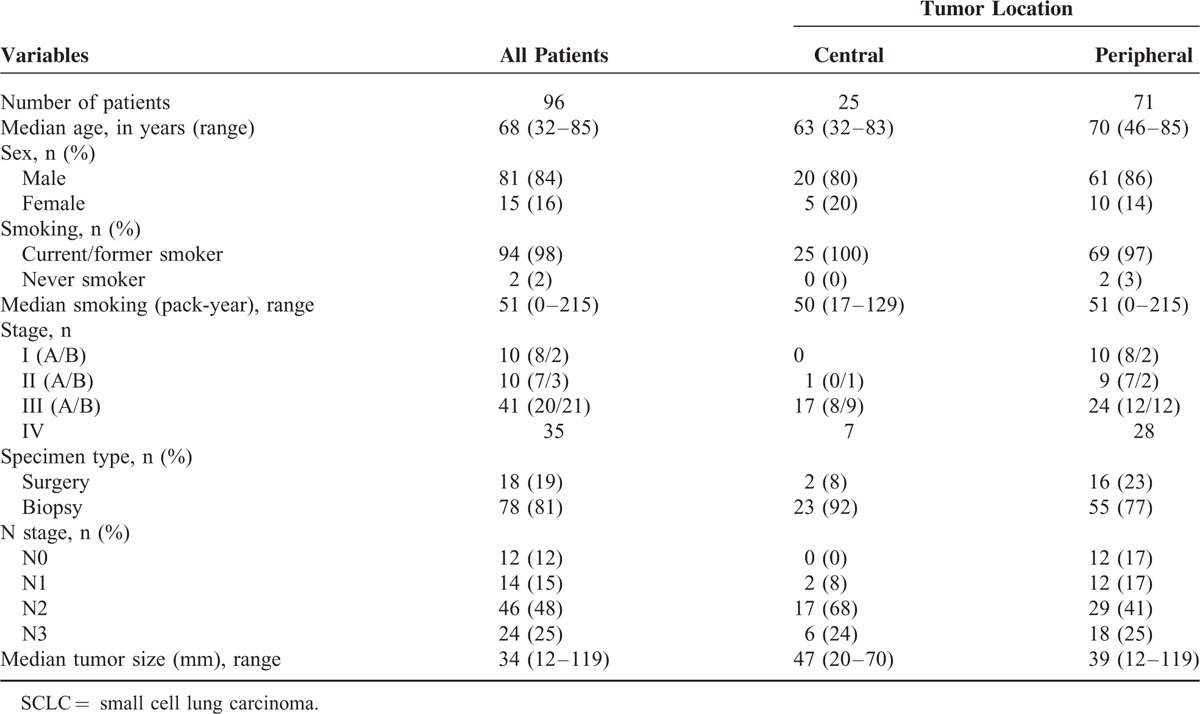
Clinicopathological Characteristics of the Examined Patients With SCLC (n = 96)

**FIGURE 3:**
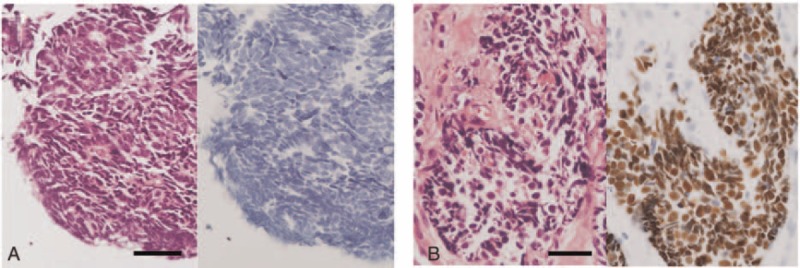
Representative HE and TTF-1 stains of a central-type (a) and a peripheral-type (b) SCLC. The scale bar represents 50 μm. SCLC =  small cell lung carcinoma, TTF-1 =  thyroid transcription factor-1.

**FIGURE 4:**
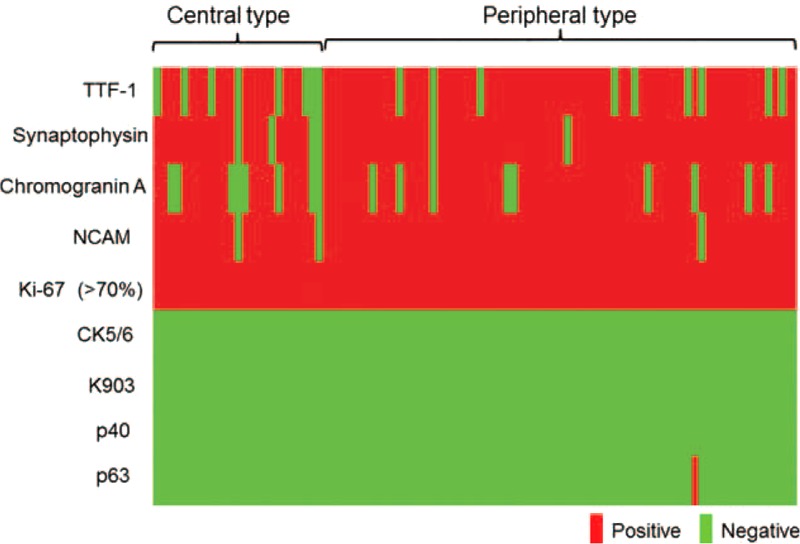
Graphical representation of immunohistochemial staining. Although there is a slight tendency that central-type tumors have less neuroendocrine marker expressions, all these tumors were typical SCLC, considering the small difference and high Ki-67 index. SCLC =  small cell lung carcinoma.

Among the 96 cases, 71 (74%) proved to be of the peripheral type, which is different from the prevailing notion that most SCLCs arise from the central region.^21,22^ As shown in Table 2, TTF-1 immunoreactivity was identified in 79/96 (82 %), among which 78% (62/79) were of the peripheral type. TTF-1 expression significantly correlated with peripheral location of the primary tumor (*P* = 0.030, chi-square test, Table 2, Figure 3). For the other tumor markers (synaptophysin, chromogranin-A, NCAM, CK5/6, K903, p40, and p63), immunohistochemical expression did not correlate significantly with location of the primary tumors (Figure 4). All the peripheral-type tumors (71/71 = 100%) and the large majority of the 25 central-type tumors (23/25 = 92%) were immunoreactive for at least 1 neuroendocrine marker (Table 2, Figure 4). In addition, the Ki-67 indices were 70% or more in all cases. There was no significant difference in clinicopathological characteristics such as age, sex, smoking, TNM stages, or specimen type (from surgery or biopsy) between central- and peripheral-type SCLCs (Table 1).

**TABLE 2:**
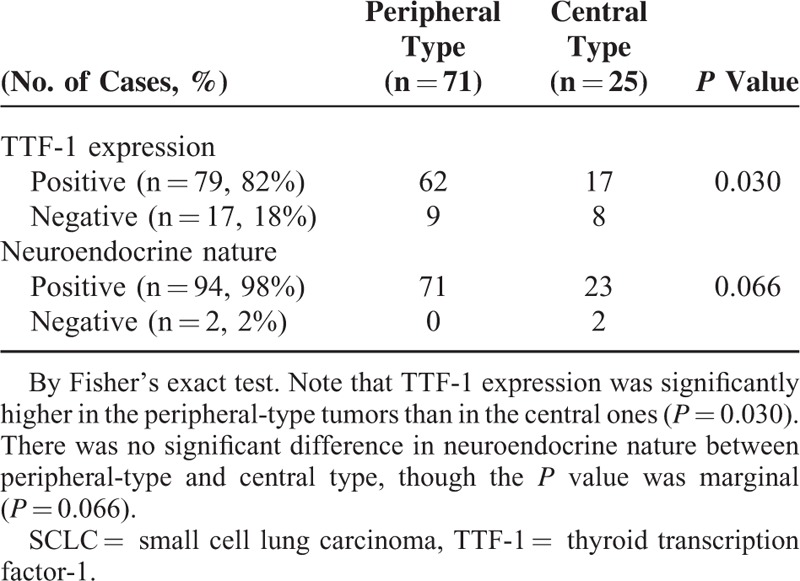
Correlation of Primary Tumor Location With TTF-1 Expression or Neuroendocrine Nature in SCLCs

**FIGURE 5:**
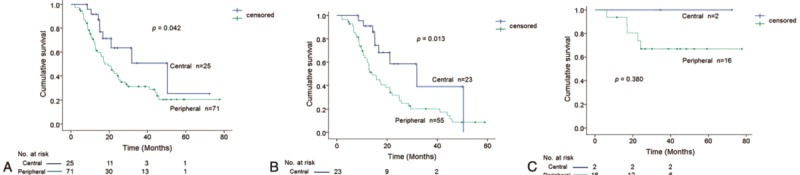
(a) Kaplan–Meier survival curves for patients with SCLC by location. A comparison between central and peripheral types shows significantly better survival in the group with central-type SCLC (n = 96, log-rank test; *P* = 0.042). (b) Biopsy cases (n = 78, log-rank test; *P* = 0.013). (c) Surgical cases (n = 18, log-rank test; *P* = 0.380). SCLC =  small cell lung carcinoma.

We examined patient survival using the Kaplan–Meier method to explore potential correlations between tumor location and TTF-1 expression. Patients with peripheral-type tumors proved to have poor survival (median overall survival: 17.5 vs 50.4 months, respectively; *P* = 0.042 by the log-rank test, Figure 5A) using both biopsy and surgical cases. Overall survival was significantly poorer for the peripheral types only for biopsy cases (n = 78, *P* = 0.013), not for surgical cases (n = 18, *P* = 0.380), when either biopsy cases or surgical cases were used, because of the small number of surgical cases (Figure 5B and C). The univariate analysis for survival with TTF-1 expression and several clinicopathologic factors indicated that sex (male), TNM stage (IIIB or IV), smoking (60 pack-years or more), specimen type (biopsy), and tumor location (peripheral) correlated significantly with prognosis (Table 3). The multivariate analysis revealed that the high TNM stages and the peripheral location were significantly unfavorable prognostic factors (*P* = 0.005, *P* = 0.015, respectively).

**TABLE 3:**
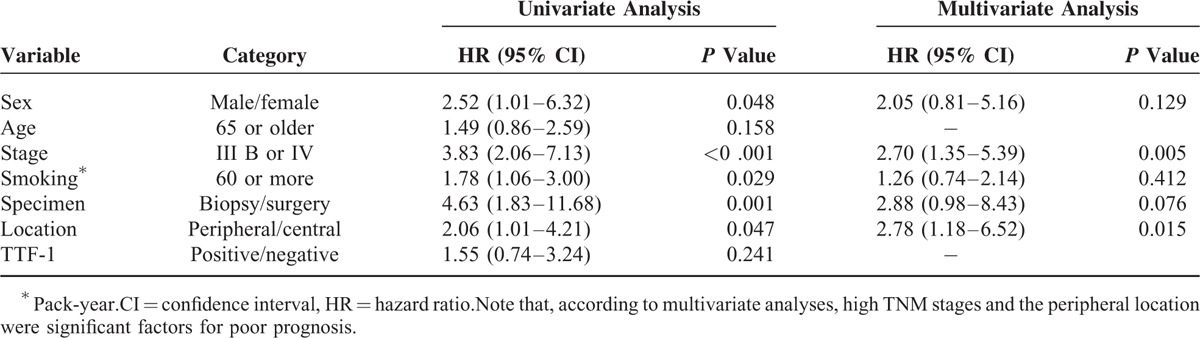
Univariate and Multivariate Analyses to Estimate Influential Factors for Prognosis Using the Cox Proportional Hazard Regression Model

## DISCUSSION

To the best of our knowledge, this study reported the correlation between primary tumor location and the TTF-1 expression in a series of nearly 100 SCLCs for the first time. Conventionally, SCLC is believed to arise mostly in the central lung, and its origin was thought to be Kulchitsky's type neuroendocrine cells existing within the bronchial mucosa. The present study clearly demonstrated that 74% of SCLCs arise in the peripheral lung and TTF-1 expression significantly correlated with peripheral location of the primary tumor. We may remember the fact that the main tumor mass certainly locates at the central area of the lung or at the mediastinum when most SCLC are first diagnosed. Without a special interest in original sites, the largest mass may be easily considered as a primary tumor. However, the original site of SCLC is quite another than the location of the largest tumor mass of SCLC diagnosed as an extensive disease. The higher frequency of central SCLC reported in the previous literature may be affected by misjudging a metastasis as an original tumor. Also, the high frequency of peripheral tumors may be explained by an ethnicity difference. Japanese may develop more tumors arising in the periphery than other ethnicity^23^ though further studies should be warranted. With regard to lung neuroendocrine tumors, the relevance of TTF-1 expression to tumor location has also been shown in carcinoids. Du et al reported that 12 of 14 TTF-1-positive pulmonary carcinoids had a peripheral location with spindle cell morphology, whereas TTF-1-negative carcinoids had a central location.^24^ This suggests that carcinoids may be classified as distinct subtypes of a central or peripheral origin, like SCLC, and that TTF-1 may be a marker for peripheral carcinoids.

Our study also showed that primary tumor location of SCLC independently correlated with prognosis. The fact that the peripheral location is an independent poor-prognostic factor suggests that the tumor nature is different between the central and peripheral origins, for example, in terms of neuroendocrine nature (Table 3), but further studies should be warranted because we have very few neuroendocrine-negative cases. Also, the tumor location may have an impact on the management of SCLC patients. Patients with peripheral SCLC may need to be treated by neo-adjuvant chemotherapy even though the tumor is stage I. Moreover, a correlation between tumor location and prognosis was found for not only SCLC but also non-SCLC tumors. Ito et al compared prognosis between central and peripheral non-SCLCs using pathologically N2 tumors (n = 40) and found that the 5-year survival rate of patients with central-type tumors was significantly better (51.5%, n = 22) than those with peripheral-type ones (21%, n = 18).^25^ More detailed examinations on histology, TTF-1 expression, and neuroendocrine nature should be performed.

Intriguingly, the latest data from a number of investigators indicate that the TTF-1 functions as a double-edged sword. Several studies have shown that positive TTF-1 expression is associated with both good prognosis and poor prognosis in lung adenocarcinoma.^16,25–29^ In addition, TTF-1 amplification is linked with poor prognosis in lung adenocarcinoma,^30,31^ and several studies suggest a possible oncogenic role of TTF-1 in lung adenocarcinoma as well as other types of cancers. Tanaka et al and Kwei et al reported that the inhibition of TTF-1 by RNA interference significantly and specifically induced growth inhibition and apoptosis in a lung adenocarcinoma cell line.^32,33^ Similarly, Homminga et al identified TTF-1 as a potential oncogene for T cell acute lymphoblastic leukemia.^34^ Ngan et al reported that a germline missense mutation of TTF-1 has been identified in families affected by multinodular goiter and papillary thyroid carcinoma.^35^ The dual faces of TTF-1 as both a pro- and an anticancer factor are complex, but these reports strongly suggest a possible oncogenic role for TTF-1 not only in lung adenocarcinoma but also in thyroid cancers and hematologic disorders. In this study, we showed that TTF-1 expression does not have prognostic relevance though correlated significantly with tumor location of SCLC. Further investigations based on molecular and cellular analyses are required to determine a role of TTF-1 in SCLC as well as in carcinoid.

The present study has several limitations of analysis and interpretation. First, this study has the difficulty of determining primary sites. We may have misjudged metastatic lesions as primary tumors. For example, when a patient presents with both an apparent intrapulmonary lesion and a more centrally located mass that looks like lymphadenopathy, it may be difficult to determine whether the intrapulmonary lesion is primary and the lymphadenopathy is a metastasis or if the lymphadenopathy-like lesion is actually a primary tumor and the intrapulmonary lesion may be a metastasis. In other words, a metastasis to hilar lymph nodes may look like a primary tumor. However, there were only 2 patients with multiple pulmonary nodules in this study, and the cases showed only 2 tumor nodules in the lung, implying that the effect of cases with multiple lesions on our results is limited. We excluded cases that were diagnosed only with cytology, and such cases may have included tumors with multiple mass lesions. Second, since our study only included the cases with biopsy materials, we were able to examine a relatively small number of cases and therefore our results may be affected by the small sample size. Our results should be confirmed in other populations and/or by further studies using a large number of cases.

In conclusion, our study confirmed that the majority of SCLCs were of the peripheral type, that the tumor location significantly correlated with TTF-1 expression and that the location was a significant prognostic factor. In addition, these results may provide interesting evidence to change the long-standing concept that SCLC is derived from cells located at the central region, such as Kultschitzky cells. Instead, the results support the hypothesis that most SCLCs are derived from TRU cells.^36^ Furthermore, in terms of the prognostic value of this study, the evaluation for primary tumor location may have a predictive role as a poor prognosis of SCLC. Further studies are warranted before SCLC can be classified into 2 subtypes with different prognosis, largely defined by tumor location.

## Supplementary Material

Supplemental Digital Content
