# Supplementary material for: Distinct Characteristics of Small Cell Lung Cancer Correlate With Central or Peripheral Origin: Subtyping Based on Location and Expression of Transcription Factor TTF-1
Source: Medicine (Baltimore). 2015 Dec 28;94(51):e2324. doi: 10.1097/MD.0000000000002324 (PMC4697988; doi:10.1097/MD.0000000000002324)
Supplement: Supplemental Digital Content [file medi-94-e2324-s001.doc]

**Suppl. Table 1.** Sources and clones of antibodies and conditions used in immunohistochemical study.

| Antibody | Source (clone) | Dilution |
| --- | --- | --- |
| TTF-1 | DAKO(8G7G3/1) | 1:1000 |
| Chromogranin A | DAKO(DAK-A3) | 1:2000 |
| Synaptophysin | Nichirei(27G12) | 1:1 |
| NCAM/CD56 | Novocastra(CD564) | 1:200 |
| CK5/6 | Merck KGaA(D5/16B4) | 1:500 |
| K903 | Enzo Life Sciences(34βE12) | 1:100 |
| p40 | Merck KGaA(1.22) | 1:800 |
| p63 | Novus(4A4) | 1:200 |
| Ki67/7B11 | Invitrogen(7B11) | 1:200 |
